# Association between high dietary intake of the *n*−3 polyunsaturated fatty acid docosahexaenoic acid and reduced risk of Crohn's disease

**DOI:** 10.1111/apt.12670

**Published:** 2014-02-24

**Authors:** S. S. M. Chan, R. Luben, A. Olsen, A. Tjonneland, R. Kaaks, S. Lindgren, O. Grip, M. M. Bergmann, H. Boeing, G. Hallmans, P. Karling, K. Overvad, S. K. Venø, F. van Schaik, B. Bueno‐de‐Mesquita, B. Oldenburg, K.‐T. Khaw, E. Riboli, A. R. Hart

**Affiliations:** ^1^ Department of MedicineNorwich Medical School University of East AngliaNorwichUK; ^2^ Norfolk & Norwich University Hospitals NHS TrustNorwichUK; ^3^ Strangeways Research LaboratoryInstitute of Public HealthUniversity of CambridgeCambridgeUK; ^4^ Institute of Cancer EpidemiologyDanish Cancer SocietyCopenhagenDenmark; ^5^ Division of Clinical EpidemiologyDKFZ‐German Cancer Research CentreHeidelbergGermany; ^6^ Department of Clinical SciencesUniversity HospitalMalmöSweden; ^7^ Department of EpidemiologyGerman Institute of Human NutritionPotsdamGermany; ^8^ Department of Public Health and Clinical MedicineNutritional ResearchUmeå UniversityUmeåSweden; ^9^ Department of Public Health and Clinical MedicineGI unitUmeå UniversityUmeåSweden; ^10^ Section for EpidemiologyDepartment of Public HealthAarhus UniversityAarhusDenmark; ^11^ Department of CardiologyAalborg University HospitalAalborgDenmark; ^12^ Department of Gastroenterology and HepatologyUniversity Medical Center UtrechtUtrechtThe Netherlands; ^13^ National Institute of Public Health and the Environment (RIVM)BilthovenThe Netherlands; ^14^ The School of Public HealthImperial College LondonLondonUK; ^15^ Division of EpidemiologyImperial College LondonLondonUK

## Abstract

**Background:**

There are plausible mechanisms for how dietary docosahexaenoic acid (DHA), an *n*−3 polyunsaturated fatty acid, could prevent Crohn's disease (CD).

**Aim:**

To conduct a prospective study to investigate the association between increased intake of DHA and risk of CD.

**Methods:**

Overall, 229 702 participants were recruited from nine European centres between 1991 and 1998. At recruitment, dietary intakes of DHA and fatty acids were measured using validated food frequency questionnaires. The cohort was monitored through to June 2004 to identify participants who developed incident CD. In a nested case–control analysis, each case was matched with four controls; odds ratios (ORs) were calculated for quintiles of DHA intake, adjusted for total energy intake, smoking, other dietary fatty acids, dietary vitamin D and body mass index.

**Results:**

Seventy‐three participants developed incident CD. All higher quintiles of DHA intake were inversely associated with development of CD; the highest quintile had the greatest effect size (OR = 0.07; 95% CI = 0.02–0.81). The OR trend across quintiles of DHA was 0.54 (95% CI = 0.30–0.99, *P*_trend_ = 0.04). Including BMI in the multivariate analysis, due to its correlation with dietary fat showed similar associations. There were no associations with the other dietary fatty acids studied.

**Conclusion:**

There were inverse associations, with a biological gradient between increasing dietary docosahexaenoic acid intakes and incident Crohn's disease. Further studies in other populations should measure docosahexaenoic acid to determine if the association is consistent and the hypothesis tested in randomised controlled trials of purely docosahexaenoic acid supplementation.

## Introduction

Crohn's disease (CD) is a chronic inflammatory bowel disease (IBD) of unknown aetiology that can affect any part of the gastrointestinal tract. Patients often have a lifelong morbidity from debilitating symptoms, require surgery and have an increased risk of intestinal failure. While genomic wide association studies have identified over 140 genetic risk loci for CD,^1^ the risk contribution from these is estimated to be less than 25%.^2^ This implies that other exposures such as environmental/lifestyle variables, including diet maybe involved in CD aetiology.^3^

The long chain dietary *n*−3 polyunsaturated fatty acids (PUFAs), docosahexaenoic acid (DHA) and eicosapentaenoic acid (EPA) may be involved in the aetiology of CD, given the importance of PUFAs in the regulation of immunological and inflammatory responses.^4^ These *n*−3 PUFAs inhibit genes that activate the inflammatory process^5^ and alter the composition of cell membranes influencing lipid raft formation in cell signalling.^6^ Furthermore, long chain *n*−3 PUFAs are metabolised to lipid mediators with weaker pro‐inflammatory properties compared to those derived from n‐6 PUFAs.^7^ More recently, emerging evidence reports that DHA and EPA can also be metabolised to lipid mediators with anti‐inflammatory and inflammation resolving properties.^8^

Despite the experimental evidence implying biological plausibility, epidemiological studies investigating associations between dietary intakes of *n*−3 PUFAs and CD aetiology are sparse. These are mainly retrospective case–control studies,^9^ and a single prospective investigation which reported the risk of developing CD was unaffected by total *n*−3 PUFAs and long chain *n*−3 PUFAs intakes.^10^ However, the effects of specific individual long chain *n*−3 PUFAs, DHA and EPA were not investigated. This is important as the biological effects of these long chain *n*−3 PUFAs and their metabolites are not uniform with them acting via both distinct and shared pathways.^11^ Accordingly, we performed this nested case–control analysis within the European Prospective Investigation into Cancer and Nutrition (EPIC) cohort investigating the effect of individual long chain *n*−3 PUFAs in CD. Demonstrating associations would imply that the dietary intake of specific *n*−3 PUFAs should be measured in future aetiological studies of CD.

## Methods

The methods of the main EPIC cohort study have been previously described.^13^ This analysis of CD is a sub‐cohort of 229 702 initially healthy men and women without CD, aged 20–74 years. Participants were resident in nine European regions, enrolled between 1991 and 1998 (Table 1). Baseline questionnaires were self‐completed by participants who supplied information on age, gender, smoking (nonsmoker, ex‐smoker or smoker at recruitment) and diet. Body mass indexes (BMI) were calculated from participants' weight and height at baseline recruitment.^14^ Habitual diet over the previous year was measured using validated country‐specific food frequency questionnaires (FFQs) consisting of approximately 200 food items and nine frequency categories of intake. Using national databases of food composition, the daily intakes of total fat, DHA (*n*−3 PUFA), EPA (*n*−3 PUFA), α‐linolenic acid (ALA, *n*−3 PUFA), linoleic acid (LA, n‐6 PUFA) and oleic acid (OA, n‐9 monounsaturated fatty acid, MUFA), total energy intake and dietary vitamin D were calculated. In all centres, the FFQs were validated against 24‐h recall questionnaires and biomarkers for specific nutrients.^15^ Intakes of ALA were recorded as this is converted to EPA and DHA.^16^ LA and OA intakes were recorded as these increase^17^ and decrease^18^ arachidonic acid mucosal concentrations, respectively, which may be involved in the inflammatory process. Dietary vitamin D was recorded as this is found in similar foods as long chain *n*−3 PUFAs and is associated with a reduced risk of CD.^19^

**Table 1:** Characteristics of the cohorts

Centre	Size of cohort	Nature of cohort	Number of participants with incident Crohn's disease (% of total cases)
Denmark (Aarhus and Copenhagen)	57 053	Population‐based cohort of men and women aged 50–64 years. Recruited between 1993 and 1997. Cases identified from national databases of inflammatory bowel disease.	11 (15.1%)
United Kingdom (Norfolk)	25 639	Population‐based cohort of men and women aged 45–74 years. Recruited between 1993 and 1997. Cases identified from follow‐up questionnaires, in‐patient admission data and histopathology records.	11 (15.1%)
Germany (Heidelberg)	25 540	Population‐based cohort of men aged 45–65 years and women aged 35–65 years. Recruited between 1994 and 1998. Cases identified from follow‐up questionnaires.	9 (12.3%)
Germany (Potsdam)	27 548	Population‐based cohort of men and women, aged 35–64 years. Recruited between 1994 and 1998. Cases identified from follow‐up questionnaires.	4 (5.5%)
The Netherlands (Amsterdam, Doetinchem, Maastricht and Utrecht)	40 092	Men & women, aged 20–70 years recruited between 1993 and 1997 from the general population of three cities (Amsterdam, Doetinchem & Maastricht) and also the breast cancer screening programme in Utrecht. Cases identified from pathology registries.	17 (23.3%)
Umeå (Sweden)	25 732	Population‐based cohort of men and women aged 45–69 years. Recruited between 1991 and 1996. Cases identified from regional databases of inflammatory bowel disease.	10 (13.7%)
Malmö (Sweden)	28 098	Population‐based cohort of men and women aged 30–60 years. Recruited between 1992 and 1996. Cases identified from regional databases of inflammatory bowel disease.	11 (15.1%)

The cohort was monitored after recruitment until June 2004 to identify initially well participants who developed a new diagnosis of CD. National disease and regional IBD registries, follow‐up questionnaires, in‐patient records and histology databases were used for case identification. Medical notes of potential cases were reviewed by local gastroenterologists to confirm diagnoses and acquire information on diagnostic investigations and disease extent. Cases of indeterminate or microscopic colitis were excluded. Those with prevalent IBD at recruitment were excluded, as were participants diagnosed with IBD <18 months after recruitment, to ensure data reflected diet prior to symptoms and diagnosis.

In a nested case–control analysis, each case was matched with four randomly selected controls matched for age at recruitment (±6 months), gender, centre and recruitment date (±3 months). Controls had to be alive on the date of diagnosis of their matched case. None of the controls had ulcerative colitis, microscopic or indeterminate colitis. Intakes of each fatty acid, total energy and vitamin D were divided into quintiles, according to the distribution across the cohort. BMI was divided into four categories (<20 kg/m^2^, 20–24.9 kg/m^2^, 25–29.9 kg/m^2^, ≥30 kg/m^2^). Baseline characteristics between cases and controls were compared using a *t*‐test for parametric distributions, a Mann–Whitney test for nonparametric ones and the χ^2^‐test for categorical ones. Odds ratios (OR) with 95% confidence intervals (CI) were calculated using conditional logistic regression (STATA version 11) for total fat, combined long chain *n*−3 PUFAs (DHA and EPA) and each individual dietary fatty acid and the development of CD, adjusted for the covariates of cigarette smoking and total energy intake. In a second analysis, total fat and combined long chain *n*−3 PUFAs were adjusted for individual relevant fatty acids due to their differing effects on the inflammatory process and interdependent metabolism, covariates in the first model and dietary vitamin D. Similar analyses were used to calculate OR for individual dietary fatty acids, adjusting for the other fatty acids that influence fatty acid metabolism and have opposing effects on inflammation. A further analysis included all the covariates from the second model plus BMI to decrease the risk of residual confounding from the likely correlation between dietary fat and BMI. OR trends were calculated across quintiles using the score 1–*n*, with *n* corresponding to quintiles 1–5 treated as continuous. In a sensitivity analysis, both the lowest and highest 2.5% of dietary nutrients were excluded as some participants may under or over report their intake. A further sensitivity analysis calculated ORs excluding those who developed CD within 3 years and 5 years after recruitment to assess if fatty acid intake is related to the time prior to diagnosis. Research protocols were approved by local ethics committees. All subjects gave written informed consent for access to their data.

## Results

A total of 73 participants developed CD (64% female) at a median age of 56.4 years (Table 2) with median time of diagnosis 5.3 years after recruitment. This equates to an appropriate incidence of 4/100 000 per year based on the size and follow‐up times of each sub‐cohort. Of those with CD, 29% had ileal disease only and 42% had colonic disease only. Cigarette smoking was positively associated with CD (OR = 2.40, 95% CI = 1.16–4.94; *P* = 0.02). The FFQ data on PUFAs were complete for 100% of cases and 99% of controls, with no significant differences between cases and controls between the median daily total energy and fatty acids (Table 2).

**Table 2:** Characteristics of cases and controls

	Controls (*n* = 292)	Cases (*n* = 73)
Age at recruitment (years, median and range)	50.2 (29.1–75.8)	50.5 (29.4–75.8)
Gender (% female)	64%	64%
Age at diagnosis (years, median and range)	–	56.4 (24.0–78.7)
Time between recruitment & diagnosis (years, median and range)	–	5.3 (1.5–14.3)
Distribution of disease (*n*, %)		
L1, ileal		21 (29%)
L2, colonic		31 (42%)
L3, ileocolonic		14 (19%)
L4, isolated upper GI disease		2 (3%)
Current smoker	27%	40%^a^
Total energy intake (kJ/day, median and range)	8486 (3684–19 722)	8711 (4509–18 056)
Body mass index (kg/m^2^) (mean, s.d.)	25.1 (3.7)	25.1 (4.0)
Median daily intakes (g/day, range)		
Docosahexaenoic acid	0.13 (0.01–1.49)	0.14 (0.02–0.97)
Eicosapentaenoic acid	0.07 (0.01–0.82)	0.06 (0.01–0.63)
α‐linolenic acid	1.30 (0.40–4.51)	1.33 (0.68–5.14)
Linoleic acid	10.33 (3.12–39.71)	10.60 (4.08–44.19)
Oleic acid	21.01 (6.05–76.75)	22.55 (10.18–62.28)

Cases were more likely than controls to be smokers.

a *P *< 0.05.

In the multivariate analysis, no differences were observed in the odds of CD across quintiles of total fat or combined long chain *n*−3 PUFAs (DHA and EPA) intake (Table 3). For individual fatty acids, adjusted for smoking and total energy intake, there were no statistically significant associations across quintiles of dietary DHA, EPA, LA, ALA or OA, although those for DHA were all less than one (Table 4). In a second analysis, including other fatty acids and dietary vitamin D, compared with the lowest quintile, all four of the higher quintiles of DHA had a reduced odds of CD, with the greatest reduction in the highest quintile (OR = 0.07, 95% CI = 0.02–0.81). There was a biological gradient across quintiles of DHA (OR_trend_ = 0.54, 95% CI = 0.30–0.99, *P*_trend_ = 0.04). No associations were seen for other the fatty acids. In particular, not adjusting for linoleic acid (n‐6 PUFA) did not alter the effect sizes in the multivariate analyses (data not shown). Including BMI did not materially alter our findings for DHA (OR_trend_ = 0.52, 95% CI = 0.28–0.96, *P*_trend_ = 0.04) or the other dietary fatty acids. The numbers according to disease site were small, but the trends across quintiles of DHA did not show any significant associations for terminal ileal or colonic disease. Excluding the lowest 2.5% and highest 2.5% of all nutrient intakes gave similar results for the highest quintile of DHA intake (OR = 0.03, 95% CI = 0.01–0.96) in the model containing all covariates. The possibility of reverse causation bias, with symptoms prior to diagnosis influencing DHA intake was also considered. However, the magnitude of the trends across quintiles for DHA were similar in the third model when excluding those diagnosed within 3 years (OR_trend_ = 0.49, 95% CI = 0.24–1.04, *P*_trend_ = 0.06) and within 5 years (OR_trend_ = 0.37, 95% CI = 0.13–1.07, *P*_trend_ = 0.07) of recruitment. This process excluded 16 and 32 cases, respectively.

**Table 3:** Dietary fats and the odds of Crohn's disease

Quintiles, g/day	Controls (*n *= 290, %)	Cases (*n* = 73, %)	OR (95% CI)^a^	OR (95% CI)^b^	OR (95% CI)^c^
Total fat					
24.4–<55.7	61 (21.0%)	12 (16.4%)	1.00	1.00	1.00
55.7–<68.9	60 (20.7%)	13 (17.8%)	1.18 (0.44–3.13)	1.18 (0.43–3.21)	1.20 (0.43–3.35)
68.9–86.9	59 (20.3%)	13 (17.8%)	1.37 (0.41–4.63)	1.31 (0.38–4.55)	1.40 (0.40–4.97)
87.6–106.7	52 (17.9%)	21 (28.8%)	2.93 (0.82–10.50)	2.68 (0.71–10.10)	2.74 (0.71–10.51)
107.3–221.1	58 (20.0%)	14 (19.2%)	1.59 (0.33–7.59)	1.48 (0.28–7.73)	1.42 (0.26–7.67)
			*P*_trend_ = 0.25	*P*_trend_ = 0.32	*P*_trend_ = 0.33
Combined long chain fatty acids (DHA and EPA)					
0.01–<0.09	56 (19.3%)	17 (23.3%)	1.00	1.00	1.00
0.09–<0.17	59 (20.4%)	13 (17.8%)	0.65 (0.27–1.57)	0.56 (0.21–1.48)	0.55 (0.21–1.47)
0.17–<0.25	60 (20.8%)	13 (17.8%)	0.64 (0.26–1.57)	0.51 (0.19–1.43)	0.52 (0.19–1.43)
0.26–<0.48	60 (20.8%)	12 (16.4%)	0.66 (0.24–1.80)	0.54 (0.17–1.72)	0.56 (0.18–1.76)
0.48–<2.15	54 (18.7%)	18 (24.7%)	1.19 (0.40–3.57)	1.03 (0.28–3.85)	1.08 (0.29–4.11)
			*P*_trend_ = 0.89	*P*_trend_ = 0.84	*P*_trend_ = 0.87

a Adjusted for smoking and total energy intake.

b Covariates in analysis 1 plus dietary vitamin D and relevant fatty acids (total fat unadjusted for individual fatty acids; total DHA and EPA adjusted for ALA, LA, OA; saturated fat adjusted for DHA, EPA, ALA, LA and OA).

c Covariates in analysis 2 and BMI.

**Table 4:** Individual fatty acids and the odds of Crohn's disease

Dietary fatty acid intake (quintiles, g/day)	Controls (*n *= 289, %)	Cases (*n* = 73, %)	OR (95% CI)^a^	OR (95% CI)^b^	OR (95% CI)^c^
Docosahexaenoic acid					
0.01–<0.07	55 (19.0%)	18 (24.7%)	1.00	1.00	1.00
0.07–<0.12	59 (20.4%)	13 (17.8%)	0.50 (0.20–1.25)	0.35 (0.10–1.26)	0.34 (0.09–1.23)
0.12–<0.17	60 (20.8%)	13 (17.8%)	0.47 (0.18–1.25)	0.22 (0.04–1.13)	0.20 (0.04–1.05)
0.17–<0.30	57 (19.7%)	15 (20.5%)	0.61 (0.22–1.68)	0.16 (0.02–1.12)	0.15 (0.02–1.09)
0.31–<1.49	58 (20.1%)	14 (19.2%)	0.49 (0.15–1.63)	0.07 (0.02–0.81)	0.06 (0.01–0.72)
			*P*_trend_ = 0.38	*P*_trend_ = 0.04	*P*_trend_ = 0.04
Eicosapentaenoic acid					
0.01–<0.03	55 (19.0%)	18 (24.7%)	1.00	1.00	1.00
0.03–<0.05	60 (20.8%)	12 (16.4%)	0.65 (0.28–1.52)	1.30 (0.42–4.00)	1.38 (0.45–4.26)
0.05–<0.09	61 (21.1%)	12 (16.4%)	0.61 (0.26–1.46)	1.55 (0.38–6.30)	1.62 (0.40–6.72)
0.09–<0.16	58 (20.0%)	14 (19.2%)	0.82 (0.31–2.15)	4.04 (0.66–24.84)	4.42 (0.68–28.70)
0.16–<0.82	55 (19.0%)	17 (23.2%)	1.04 (0.36–2.96)	6.43 (0.72–57.90)	8.56 (0.89–83.82)
			*P*_trend_ = 0.99	*P*_trend_ = 0.11	*P*_trend_ = 0.09
α‐linolenic acid					
0.40–<0.94	59 (20.4%)	14 (19.2%)	1.00	1.00	1.00
0.94–<1.16	60 (20.8%)	12 (16.4%)	0.64 (0.24–1.70)	0.38 (0.12–1.19)	0.41 (0.13–1.30)
1.16–<1.44	54 (18.7%)	19 (26.0%)	1.17 (0.47–2.88)	0.71 (0.24–2.12)	0.76 (0.25–2.30)
1.44–<1.88	59 (20.4%)	13 (17.8%)	0.67 (0.23–1.98)	0.33 (0.08–1.28)	0.32 (0.08–1.30)
1.90–<5.14	57 (19.7%)	15 (20.5%)	0.73 (0.20–2.67)	0.38 (0.07–1.98)	0.40 (0.07–2.09)
			*P*_trend_ = 0.85	*P*_trend_ = 0.35	*P*_trend_ = 0.34
Linoleic acid					
3.12–<6.93	62 (21.5%)	11 (15.0%)	1.00	1.00	1.00
6.93–<9.41	55 (19.0%)	17 (23.2%)	1.62 (0.67–3.90)	2.42 (0.87–6.77)	2.36 (0.83–6.69)
9.44–<11.35	57 (19.7%)	16 (22.0%)	1.72 (0.65–4.55)	2.47 (0.75–8.11)	2.42 (0.73–8.06)
11.35–<15.03	61 (21.1%)	11 (15.0%)	0.94 (0.32–2.76)	1.39 (0.40–4.84)	1.32 (0.38–4.65)
15.03–<44.20	54 (18.7%)	18 (24.7%)	1.91 (0.67–5.46)	2.34 (0.65–8.42)	2.34 (0.62–8.19)
			*P*_trend_ = 0.57	*P*_trend_ = 0.61	*P*_trend_ = 0.66
Oleic acid					
6.05–<14.96	61 (21.1%)	12 (16.4%)	1.00	1.00	1.00
14.98–<18.62	58 (20.1%)	14 (19.2%)	1.22 (0.48–3.13)	1.43 (0.51–4.00)	1.29 (0.45–3.67)
18.69–<23.93	58 (20.1%)	15 (20.5%)	1.29 (0.44–3.79)	1.52 (0.43–5.36)	1.61 (0.44–5.89)
24.01–<30.58	55 (19.0%)	17 (23.3%)	1.75 (0.54–5.62)	2.04 (0.51–8.08)	2.04 (0.50–8.34)
30.80–<76.75	57 (19.7%)	15 (20.5%)	1.39 (0.31–6.17)	1.94 (0.35–10.64)	2.04 (0.35–11.87)
			*P*_trend_ = 0.49	*P*_trend_ = 0.37	*P*_trend_ = 0.35

a Adjusted for smoking and total energy intake.

b Covariates in analysis 1, the other four dietary fatty acids and dietary vitamin D.

c Covariates in analysis 2 and BMI.

## Conclusions

The main finding of this study was a statistically significant inverse association between the development of incident CD and the dietary intake of the long chain *n*−3 PUFA, DHA. The highest quintile of DHA was associated with a reduction in the odds of developing incident CD of 94%, the equivalent of eating one to two portions of oily fish per week. The data suggest that long‐term DHA intake may be important as the effect sizes were similar across varying time periods before diagnosis. Evidences to support a true aetiological association with DHA are: plausible biological mechanisms, a large effect size and a biological gradient with increasing DHA dietary intakes. Mechanistic studies suggest that the anti‐inflammatory properties of DHA may be partly mediated via competitive inhibition of arachidonic acid metabolism,^20^ which is required for the production of pro‐inflammatory lipid derived mediators (eicosanoids).^22^ These include leukotrienes and prostaglandins that are present in increased amounts in the mucosa of patients with CD.^23^ DHA also inhibits key transcription factors such as PPARγ and NFκΒ, required for the intracellular signalling cascade that activates inflammation.^5^ More recently, studies have reported that DHA is metabolised to resolvins, lipid mediators with both anti‐inflammatory and inflammation resolving properties. These may have a role in CD aetiology as resolvins prevent gastrointestinal inflammation in murine models of CD.^24^ For EPA, the lack of inverse associations, but nonstatistically significant positive ORs were completely unexpected, as experimental work has reported that EPA is metabolised to lipid mediators with similar anti‐inflammatory properties to DHA.^25^ The reasons for this are unknown, although hypothetically, the greater range of dietary intakes for DHA compared to EPA may have made associations for DHA easier to detect. However, importantly some studies have reported that EPA is less effective than DHA in suppressing inflammation^26^ and may actually be pro‐inflammatory.^27^ This may result from EPA being metabolised to leukotriene B_5_, which has weak pro‐inflammatory activity^7^ and prostaglandin E_3_, which disrupts the intestinal epithelial barrier.^28^ Moreover, while one would assume that if DHA and EPA were present in identical food sources (i.e. oily fish), and were predominantly anti‐inflammatory, then the directions of the associations for both *n*−3 PUFAs would be the same. However, that EPA but not DHA is also present in spreads and cooking oils may also help explain the discrepancy as both these *n*−3 PUFAs are not obtained from completely identical sources. Accordingly, the role of these two *n*−3 PUFAs needs to be clarified by following our cohort further to identify more cases to improve the precision of effect sizes and by investigating *n*−3 PUFAs in other epidemiological work. Notably, work on ulcerative colitis in this cohort reported similar inverse associations between higher dietary intakes of DHA, but not EPA.^29^

The inverse association between DHA and CD was only observed following adjustment for fatty acids and vitamin D that could all potentially influence the biological effects of DHA and EPA. Adjustments were performed as the different fatty acids influence firstly the metabolism of each other and secondly the inflammatory pathway itself. As described earlier, dietary ALA is converted to EPA and DHA^16^ while LA and OA increase^17^ and decrease^18^ mucosal arachidonic acid concentrations, respectively, which is metabolised to pro‐inflammatory mediators. The fact that combined long chain *n*−3 PUFAs (DHA and EPA) did not affect the odds of CD is consistent with the only other prospective analysis performed in the exclusively female Nurses' Health Study Cohort,^10^ which did not investigate individual long chain *n*−3 PUFAs. To the best of our knowledge, our study is the first prospective study of CD investigating DHA and EPA separately, which warrants confirmation in other populations.

Evidence supporting a beneficial effect of DHA in preventing CD would be provided from randomised controlled trials (RCTs) of DHA supplementation in the general population investigating if this reduced incidence. Clearly, these are impractical due to the large number of participants needed to accrue sufficient cases. DHA in the form of fish oil supplementation has been assessed in RCTs of preventing relapse in patients with CD,^30^ although subsequent meta‐analyses reported little benefit.^32^ This may be as a consequence of *n*−3 PUFAs having different roles in CD aetiology compared to the natural history in patients with established disease. Alternatively, given that fish oil supplements contains both DHA and EPA, which may have different biological effects, perhaps dietary interventions should focus on increasing just DHA intake alone.

Our study methodology had several strengths including the prospective collection of dietary information which minimised recall biases. Selection biases were reduced as cases and controls were drawn from the same population. We considered the potential confounding effect of smoking, but had no information on other covariates such as family history of CD or appendicectomy. Residual confounding is a possibility if DHA is a marker for another, possibly dietary exposure, which is actually the true aetiological factor. However, including dietary vitamin D in our models, which is found in similar foods to longer chain *n*−3 PUFAs, and BMI, did not change our results. Similarly, the effect sizes were unaffected when adjustments for linoleic acid (n‐6 PUFA) were excluded suggesting that the effects of low DHA intake were not as a consequence of higher n‐6 intakes. Other aetiological hypotheses will be explored in future work and DHA adjusted for any associations. Aspects of our study are generalizable in that our cohort included both genders recruited from several countries with the number developing CD approximately similar to that expected from data in a large European study of IBD incidence.^33^ The anatomical distribution of CD is probably explained by the older age of our cohort as these patients are more likely to develop colonic disease.^34^ We recruited mainly middle aged to elderly participants and so were unable to ascertain if our findings apply to younger people. However, UK studies reporting that consumption of oily fish, a marker of DHA intake is up to 30% lower in those aged 19–64 years, compared to older people.^35^ Therefore, the benefits of increasing DHA intake may be greater in younger persons. A limitation is measurement error inherent in the FFQs for recording habitual diet. Although FFQs are pragmatic to use in large epidemiological studies, they are less accurate than either food diaries or weighed records.^36^ Furthermore, we only had one measure of diet at recruitment, although it has been reported that diet remains relatively stable over time in adults in terms of categories of nutrient intake.^37^ Both these sources of measurement error would result in an underestimate of effect sizes rather than a spurious overestimate. A further limitation is the number of CD cases in our study may mean that smaller dietary associations would be undetected.

Finally, our statistical analysis, namely adjusting for all the fatty acids, has both strengths and limitations. The analysis was an *a priori* one, which we decided was important as fatty acids can firstly influence the inflammatory process in different ways and secondly affect the metabolism of each other. Therefore, failure to consider each one in the analysis could mean effects went undetected or were spuriously exaggerated. However, we acknowledge that adjusting for multiple nutrients can introduce statistical instability leading to imprecise measures of the effect sizes. Furthermore, including several factors in a model correlated with each other, as occurred for DHA and EPA (*r* = 0.95), can result in collinearity, although as discussed previously, there were some differences in the food sources of these. This phenomenon may introduce spurious inaccurate estimates of the individual effect sizes, although the predictivity of the model as a whole remains accurate. If collinearity is the explanation, then in our adjusted analysis, this means that the effect of DHA is dependent on the other fatty acids included. Despite the possibility of collinearity, evidence for a true effect of DHA are: a plausible biological mechanism supported by the inverse direction of the associations, a dose–response effect, and in the model which excluded other fatty acids, the associations for quintiles were all in the same direction as the model in which they were included. Whether the inverse association with DHA is real, can only be clarified in randomised controlled trials in participants who are given purely DHA supplementation. Importantly, additional EPA should not be administered in view of the suggestive positive trend we observed and as previous trials of fish oil supplementation,^31^ which contained both DHA and EPA did not report any clinical benefit.

In conclusion, we report an inverse association between higher intakes of dietary DHA and the development of CD. Evidence for a causal association is supported by plausible biological mechanisms, large effect sizes and a possible dose–response relationship. To confirm causality, consistent findings are needed from other populations and laboratory studies on potential biological mechanisms. While appreciating that DHA may play different roles in CD aetiology and treatment, the hypothesis of a protective effect of DHA would be supported by good quality interventional studies of DHA specifically in treating relapses in patients. These trials would assess the effect of the dose and timing of DHA administered, compared to appropriate placebos, and would also remove the potential problem of collinearity which can exist in observational studies. Such work is important, for if our findings are consistent then the incidence of CD may be reduced by dietary modifications and interventions.

## Authorship

*Guarantor of the article*: Simon Chan.

*Author contributions:* SSMC and ARH designed the study, recruited the centres, analysed the data and wrote the paper. RL generated the master dataset, performed data entry, provided support on statistical analysis and contributed to writing the paper. The remaining co‐authors AO, AT, RK, SL, OG, MMB, HB, GH, PK, KO, SKV, FvS, BB, BO, KK and ER are principal investigators in their respective centres who contributed to the local design, development and recruitment of participants into their cohorts. These authors generated the local IBD databases, and contributed to the analysis and writing of the manuscript. All authors approved the final version of the manuscript.

## Supplementary Material

**Table S1**. Dietary fats and the odds of Crohn's disease.
