# Supplementary material for: Association between high dietary intake of the n−3 polyunsaturated fatty acid docosahexaenoic acid and reduced risk of Crohn's disease
Source: Aliment Pharmacol Ther. 2014 Feb 24;39(8):834–42. doi: 10.1111/apt.12670 (PMC4114542; doi:10.1111/apt.12670)
Supplement: Supplementary file 1 — Table S1. Dietary fats and the odds of Crohn's disease. [file apt-39-834-s1.pptx]

## Slide 1
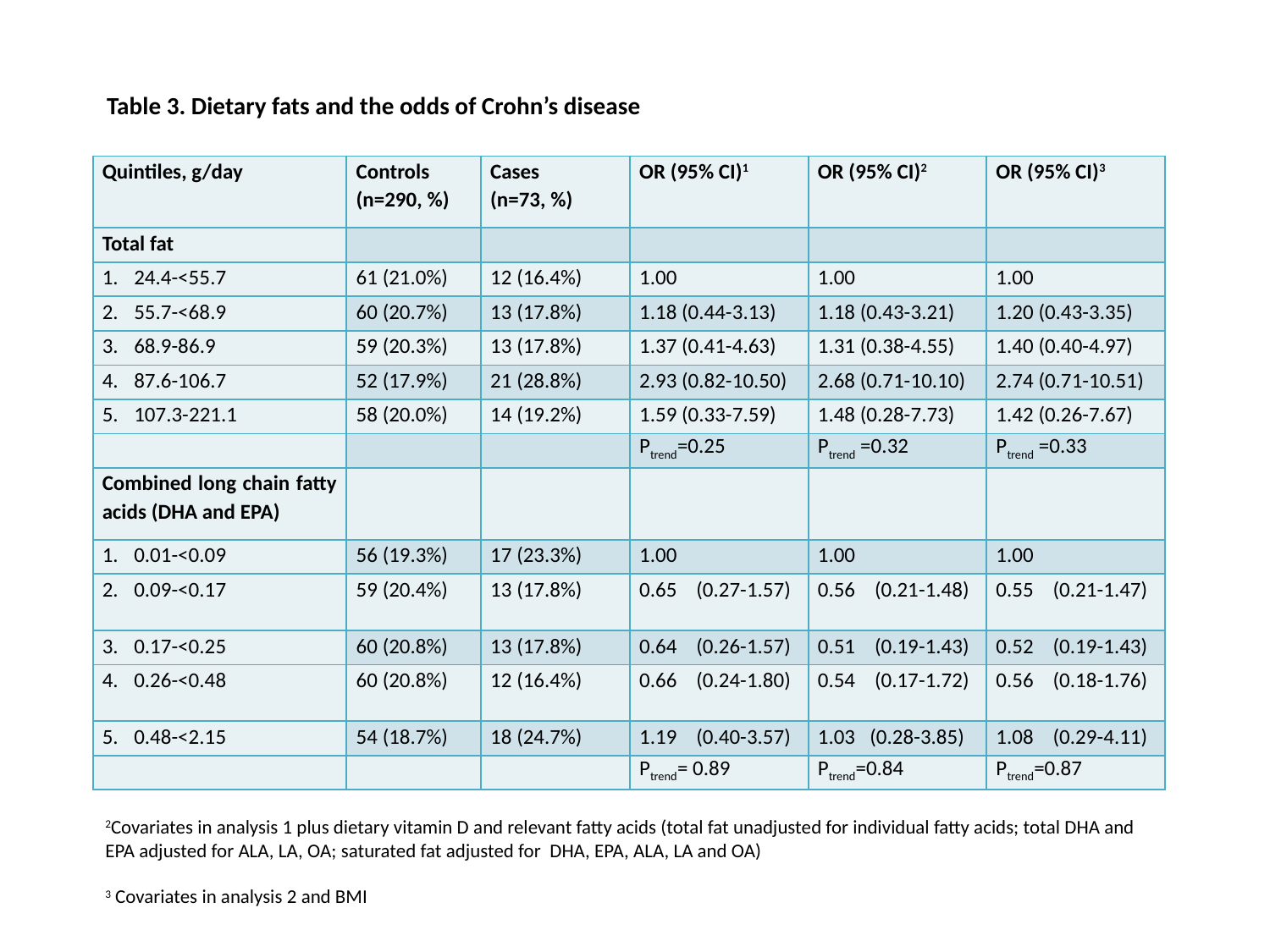

Table 3. Dietary fats and the odds of Crohn’s disease
| Quintiles, g/day | Controls (n=290, %) | Cases (n=73, %) | OR (95% CI)1 | OR (95% CI)2 | OR (95% CI)3 |
| --- | --- | --- | --- | --- | --- |
| Total fat | | | | | |
| 24.4-<55.7 | 61 (21.0%) | 12 (16.4%) | 1.00 | 1.00 | 1.00 |
| 55.7-<68.9 | 60 (20.7%) | 13 (17.8%) | 1.18 (0.44-3.13) | 1.18 (0.43-3.21) | 1.20 (0.43-3.35) |
| 68.9-86.9 | 59 (20.3%) | 13 (17.8%) | 1.37 (0.41-4.63) | 1.31 (0.38-4.55) | 1.40 (0.40-4.97) |
| 87.6-106.7 | 52 (17.9%) | 21 (28.8%) | 2.93 (0.82-10.50) | 2.68 (0.71-10.10) | 2.74 (0.71-10.51) |
| 107.3-221.1 | 58 (20.0%) | 14 (19.2%) | 1.59 (0.33-7.59) | 1.48 (0.28-7.73) | 1.42 (0.26-7.67) |
| | | | Ptrend=0.25 | Ptrend =0.32 | Ptrend =0.33 |
| Combined long chain fatty acids (DHA and EPA) | | | | | |
| 0.01-<0.09 | 56 (19.3%) | 17 (23.3%) | 1.00 | 1.00 | 1.00 |
| 0.09-<0.17 | 59 (20.4%) | 13 (17.8%) | 0.65 (0.27-1.57) | 0.56 (0.21-1.48) | 0.55 (0.21-1.47) |
| 0.17-<0.25 | 60 (20.8%) | 13 (17.8%) | 0.64 (0.26-1.57) | 0.51 (0.19-1.43) | 0.52 (0.19-1.43) |
| 0.26-<0.48 | 60 (20.8%) | 12 (16.4%) | 0.66 (0.24-1.80) | 0.54 (0.17-1.72) | 0.56 (0.18-1.76) |
| 0.48-<2.15 | 54 (18.7%) | 18 (24.7%) | 1.19 (0.40-3.57) | 1.03 (0.28-3.85) | 1.08 (0.29-4.11) |
| | | | Ptrend= 0.89 | Ptrend=0.84 | Ptrend=0.87 |
1Adjusted for smoking and total energy intake
2Covariates in analysis 1 plus dietary vitamin D and relevant fatty acids (total fat unadjusted for individual fatty acids; total DHA and EPA adjusted for ALA, LA, OA; saturated fat adjusted for DHA, EPA, ALA, LA and OA)
3 Covariates in analysis 2 and BMI
